# Could CT screening for lung cancer ever be cost effective in the United Kingdom?

**DOI:** 10.1186/1478-7547-6-5

**Published:** 2008-02-26

**Authors:** David K Whynes

**Affiliations:** 1 Professor of Health Economics, School of Economics, University of Nottingham, Nottingham, NG7 2RD, UK

## Abstract

**Background:**

The absence of trial evidence makes it impossible to determine whether or not mass screening for lung cancer would be cost effective and, indeed, whether a clinical trial to investigate the problem would be justified. Attempts have been made to resolve this issue by modelling, although the complex models developed to date have required more real-world data than are currently available. Being founded on unsubstantiated assumptions, they have produced estimates with wide confidence intervals and of uncertain relevance to the United Kingdom.

**Method:**

I develop a simple, deterministic, model of a screening regimen potentially applicable to the UK. The model includes only a limited number of parameters, for the majority of which, values have already been established in non-trial settings. The component costs of screening are derived from government guidance and from published audits, whilst the values for test parameters are derived from clinical studies. The expected health gains as a result of screening are calculated by combining published survival data for screened and unscreened cohorts with data from Life Tables. When a degree of uncertainty over a parameter value exists, I use a conservative estimate, i.e. one likely to make screening appear less, rather than more, cost effective.

**Results:**

The incremental cost effectiveness ratio of a single screen amongst a high-risk male population is calculated to be around £14,000 per quality-adjusted life year gained. The average cost of this screening regimen per person screened is around £200. It is possible that, when obtained experimentally in any future trial, parameter values will be found to differ from those previously obtained in non-trial settings. On the basis both of differing assumptions about evaluation conventions and of reasoned speculations as to how test parameters and costs might behave under screening, the model generates cost effectiveness ratios as high as around £20,000 and as low as around £7,000.

**Conclusion:**

It is evident that eventually being able to identify a cost effective regimen of CT screening for lung cancer in the UK is by no means an unreasonable expectation.

## Background

Lung cancer has long been one of the principal causes of cancer death in industrialised countries. In the 1950s, the availability of radiography led physicians to believe that screening for the disease by means of regular chest X-ray and sputum cytology was both possible and desirable. In the USA, guidance to this effect was issued and clinical trials were initiated. However, once it had been appreciated that the survival gains anticipated in theory were not being realised in practice, screening began to lose its appeal [1]. Interest in screening has since been re-awakened, as a result of both developments in imaging technologies (notably low-dosage, spiral computed tomography, CT) and novel cancer treatments which promise improved survival following diagnosis. Clinical studies of CT screening have been published but, to date, none has had a comparator group, nor have mortality improvements as a direct consequence of screening been demonstrated. No randomised controlled trial (RCT) has yet reported, although European trials have been initiated [2,3].

Compared with the enthusiasm expressed in the 1950s and 1960s, the current advocacy for lung cancer screening is being tempered with caution [4-6]. In the USA in particular, practitioners are being urged to await the results of RCTs prior to implementing CT screening regimens which could prove both ineffective and costly [7-10]. In the UK in 2006, a review of CT screening conducted for the Health Technology Assessment (HTA) Programme concluded that there existed insufficient evidence to demonstrate either clinical or cost effectiveness in the UK setting. To fill the evidence gap, the review advocated both the initiation of UK-centred trials of CT screening and further research into lung cancer aetiology, quality of life and related resource use [11].

Collecting evidence by RCT is costly and time-consuming. In the UK, formal health technology assessment influences public health care allocation decisions, and it is unlikely that CT screening for lung cancer would be implemented in the absence of a demonstration of its cost effectiveness. By the same token, however, a potential sponsor of research in the UK would be disinclined to allocate funds to evaluate screening, unless it was reasonably confident that a practical screening programme would result. Lung cancer researchers thus find themselves in a position not unfamiliar to medical researchers more generally, namely, one of being unable to prove cost effectiveness without having first conducted a trial, whilst being unable to secure financial support for a trial without having first demonstrated cost effectiveness. Modelling can contribute to breaking the circular logic, by establishing whether or not the existing evidence precludes the possibility of a programme ever being cost effective.

### Existing models

The 2006 HTA review identified six economic evaluation models of CT screening constructed by Japanese or US researchers [12-17]. Since the review, an additional Australian study has been published [18]. All of the modelled screening regimens were hypothetical, although two [12,17] were based on experimental protocols. Six out of seven of the models produced incremental cost-effectiveness ratios (ICERs) for screening, expressed in terms of costs of either expected life years (LYs) or expected quality-adjusted life years (QALYs) gained. Each model embodied a sensitivity analysis, to demonstrate the consequences of varying the model's assumptions, and the ICER results were presented as ranges. Across the studies, the lowest ratio between minimum and maximum ICERs presented was 3.3 [16], whilst the highest was 20.0 [14].

In the UK, the National Institute for Health and Clinical Excellence (NICE) considers evidence of both clinical and cost effectiveness when deciding on whether or not to sanction the introduction of new NHS treatments or services. According to NICE, interventions with prospective ICERs below £20,000 per QALY can be accepted as cost effective, although all those with ICERs up to around £30,000 merit consideration [19]. The estimated ICERs from the screening models can be translated into £-sterling at current prices, using both the purchasing power parity exchange rate at the reference date of each study and the National Health Service (NHS) HCHS Pay and Price Index. After so doing, it emerges that, for two of the models [15,17], the lower end of the ICER range is below £5,000. Indeed, the £30,0000 NICE threshold appears to be outside the range of only one of the models [14].

Whilst it might be supposed that this finding strengthens the case for the cost effectiveness of a UK programme, there are grounds for distrusting inferences from the earlier models. First, the HTA review describes the quality of reporting in the studies as "poor", noting that "replication and verification by the reader is not possible, as the inner workings of the models are not disclosed" [[11] p.25]. The lack of transparency precludes the assessment of scientific quality, so that accepting the models' results as being authoritative requires a considerable act of faith. Second, the wide range of ICERs produced by the different models is symptomatic of the absence of scientific evidence on major aspects of the disease and its management. With primary data being unavailable, all of the models have been driven by assumptions about lung cancer aetiology, disease progression, management protocols, screening and treatment effectiveness, survival and the like. Most of these assumptions are "uncorroborated" [[11] p.41]. Variability in results is compounded in models based on stage transition and sequencing, because later calculations rest on the assumptions behind earlier ones.

Third, it is often felt that, because diseases are complex, models too must be complex, embodying a large number of parameters and linkages between them. However, when scientific facts are in short supply, a larger number of parameters increases the extent of under-identification. Parameter values have to be assigned by assumption or by guesswork, and a model which requires a larger number of such guesses generates simultaneously a larger number of permutations of values, all of which will yield solutions consistent with those few observations which actually do exist. Thus, over-complex models generate wide ranges of possible results, with no guidance as to those which are the more probable.

Finally, and of considerable significance to the question of cost effectiveness in a UK setting, none of the published models has been populated with UK data. Whilst the use of non-UK data might be tolerable with respect to certain epidemiological parameters, such as aetiology and risk, clinical practices vary between countries. Moreover, the costs of labour, equipment, medicines, etc. are likely to be country- or system-specific [20]. Using only currency exchange rates, the costs of interventions assessed in other countries are unlikely to translate accurately as measures of resource use in the NHS environment.

I conclude that making inferences from earlier models is insufficient to answer the question of whether or not UK screening could be cost effective. Accordingly, I adopt a more direct approach and model a possible UK programme. The model, which is based on one originally developed for colorectal cancer [21], addresses the cost effectiveness of the screening programme per se, as opposed to that of a programme consequent upon an empirically-unsupportable disease progression model. I employ transparent linear algebra, as opposed to opaque simulation and, wherever possible, include only parameters whose values can be established scientifically. When recourse to assumption or guesswork is unavoidable, I choose values least conducive to making screening appear cost effective.

## Method

An actual protocol for a UK screening programme remains a matter of conjecture. For example, would screening be "once-only" or repeated? If the latter, what would be the inter-round time interval? The defining characteristics of the target population also remain to be decided although, for reasons which will become apparent shortly, risk of disease will certainly be relevant. As the closest approximation to a future programme, I model a screening protocol based on the National Institute for Health and Clinical Excellence's current guidance for managing patients with suspected lung cancer [22]. I presume that all individuals in a cohort targeted for screening receive a CT scan, and those with negative results thereafter exit the programme. Individuals recording positive results will be investigated further, to filter out from eventual treatment those whose test results are false-positive. Patients with suspected central lesions will be investigated by bronchoscopy, whilst those with peripheral lesions will receive a percutaneous transthoracic needle biopsy. Other diagnostic options, such as sputum cytology or positron emission tomography (PET) scanning, will be reserved for cases where either bronchoscopy or needle biopsy is deemed impracticable. Subjects in whom cancer is confirmed will proceed to treatment.

The costs of the screening programme additional to the costs resulting from symptomatic presentation are therefore (i) the costs of CT-testing all individuals in the screening cohort, plus (ii) the costs of investigating all CT-positives, plus (iii) the costs of treating the true-positives, minus (iv) the costs of confirming and treating cancer amongst those who, in the absence of screening, would have presented symptomatically. The benefits of screening – a longer life expectancy as a result of cancers being detected and treated earlier than would have occurred otherwise – are confined to those individuals who record true-positive results.

In the remainder of the Methods section, all of the model's parameters and functional forms are defined, and the baseline values are assigned. For convenience, the definitions of the variables, their base values and the sources are summarised in Table 1.

**Table 1:** Glossary of model parameters

	***Definition***	***Baseline value & source***
*A*	Age – sub-scripted CT (age at CT screening) and SP (age at symptomatic presentation)	
*B*	Health benefit gained as a result of early detection, per cancer	1.7 QALYs – determined by the survival model
*C*	Probability of an individual of screening age surviving until symptomatic presentation	0.88 – UK Life Tables [38]
*E*	Lead time (years between detection at screening and symptomatic presentation)	8 years – [44, 45]
*G*_*CT*_	Gross costs of screen-detecting and treating a case of lung cancer	£12,000 – [26, 27]
*G*_*SP*_	Gross costs of diagnosing and treating a cancer presenting symptomatically	£7,050 – [26, 27, 29, 30]
*I*	Cost of investigating a positive resulting from the initial screening test	£503 – Cost of bronchoscopy [23]
*M*_*A*_	Mortality rate at age *A*	
*N*	Numbers of individuals in a cohort – sub-scripted CT (screening) and SP (symptomatic presentation)	
*P*	Prevalence of lung cancer in the population targeted for screening	1% – average of [34-36]
*R*	Discount rate	3 1/2% – [46]
*S*	Unit cost of the initial screening test (CT scan).	£60 – [23]; includes £4 allowance for administration
*T*	The net additional cost of treating a screen-detected cancer, as opposed to one presenting symptomatically	£7,286 – calculated from *C*, *E*, *G*_*CT*_, *G*_*SP *_and *R*
*X*	Sensitivity of the screening test	85% – [11, 14, 31]
*Y*	Specificity of the screening test	85% – [11, 31, 32]

### Model structure

• All costs and outcomes subsequently defined are additional to those which would accrue to the management of an unscreened cohort of equal size.

• The screening programme requires each of *N *subjects to take a screening test, at a cost of *S *per subject. Test sensitivity, specificity and disease prevalence are *X*, *Y *and *P*, respectively. Those recording negative test results exit the programme, whilst those recording positive results will be investigated further, at unit cost *I*.

• For the cohort, the expected number of true positive test results = *NPX*, whilst the expected number of false positives = *N*(1-*P*)(1-*Y*). We assume that the investigation is definitive, i.e. always yields the correct diagnosis.

• The expected cost of screening *N *subjects and of detecting the cancers equals the costs of the tests undertaken, plus the costs of investigating the positives, both true and false:

*NS *+ *NPXI *+ *N*(1-*P*)(1-*Y*)*I*

• Detected cancers will be treated, and screening influences treatment in three ways. First, in comparison with symptomatic presentation, screen-detection moves the time of treatment forward, i.e. treatment costs are incurred earlier. Second, as the cohort ages, some of the individuals who record true positives at the time of screening, and who are treated accordingly, would have died of other causes before presenting with symptoms, had screening not been available. These subjects would then have required no cancer-specific treatment. Third, to be successful, screening will change the stage distribution of identified disease in favour of earlier stages, and the costs of early-stage treatment may differ from those of late-stage. The net additional cost of treating each cancer detected by screening, *T*, will therefore be governed by (i) the lead time which, coupled with the interest rate, determines the degree of discounting on costs which are incurred in the future, (ii) the probability of subjects with undetected cancers dying before presentation, (iii) the costs of treating both screen-detected and symptomatically-presenting cancers. The gross costs of diagnosing and treating a screen-detected cancer are represented as *G*_*CT*_, and those of a symptomatically-presenting cancer as *G*_*SP*_. The time elapsing between screen detection and symptomatic presentation is *E*, the discount rate is *R*, and the probability of an individual of screening age surviving until presentation is *C*. Then:

$$T=G_{CT}-\frac{CG_{SP}}{{\left(1+R\right)}^{E}}$$

• Confirmed false positives on investigation exit the programme, incurring no further costs. The total costs of the screening programme additional to no screening, i.e. test costs, investigation costs, and the extra treatment costs of the true positives, are therefore:

*NS *+ *NPX*(*I *+ *T*) + *N*(1-*P*)(1-*Y*)*I*

• As a result of screen-detection and early-stage treatment, each true positive gains a benefit, *B*, measured in life years (LYs) or quality-adjusted life years (QALYs). The total expected health gain for the screening programme is therefore:

*NPXB*

• The ICER for the screening regimen is equation (3) divided by equation (4). Further division of both numerator and denominator by *N *produces:

$$ICER=\left[\frac{S+PX\left(I+T\right)+\left(1-P\right)\left(1-Y\right)I}{PXB}\right]$$

• The numerator of equation (5) is the expected cost of the screening regimen per person screened, whilst the denominator is the expected benefits of screening per person screened.

### Unit costs

In England, the ostensible costs of many clinical procedures are presented as tariffs. Tariffs are based on cost estimates routinely collected from individual health care providers, each of which is expected to employ a standard template for recording resource use. The variation in unit costs across providers is typically wide and, as collection is far from transparent, it is not evident that tariff-based costs necessarily reflect true economic costs. However, tariffs are intended to form the foundation of National Health Service (NHS) accounting, so the use of tariff-costs in a model of a potential NHS screening programme would seem appropriate. The tariff for a CT scan is £56 [23], although any future mass screening programme will require an administrative structure. Some ten years ago, it was estimated that the English cervical screening service expended £6 million on administering the screening of over 3 million women each year [24]. To allow for the cost of administration, therefore, I add £4 to the cost of each scan and set the unit cost of the CT screening test at £60. This cost (and all subsequent costs) is expressed in 2004 prices.

Of the investigation alternatives under consideration, only PET is more costly than bronchoscopy, although PET scanning would be reserved for the minority of cases where other diagnostic methods would fail. A UK study [25] has suggested that bronchoscopy is likely to be appropriate in the majority of cases. For my model, therefore, I assume that all screening subjects with positive CT results will be investigated by bronchoscopy, an assumption which appears likely to overstate the expected unit costs of investigation. According to the NHS tariff, the unit cost of bronchoscopy is £503 [23].

The costs of treating lung cancer are not clear-cut, as the national tariff describes unit costs for procedures rather than for patients. Any patient entering treatment might require more than one procedure and it is likely that few patients will undergo precisely the same treatment path following diagnosis. In theory, therefore, the range of potential treatment costs is extremely wide. For model purposes, I employ treatment cost estimates derived from two empirical studies, each of which employed an audit, as opposed to a tariff, approach. These studies calculated patient-specific costs for 253 patients in the Trent region [26] and for 109 patients in Newcastle [27], each managed over a maximum of 4 years. Expressed at 2004 prices, the studies produced mean treatments costs of approximately £8,800 and £14,200 per case, respectively. The higher costs in the second study appear to be attributable to longer mean lengths of hospital stay during each of the various treatment phases. I accordingly choose an intermediate value of £12,000 per case to represent the gross cost of treating a screen-detected cancer.

Lung cancers in the UK are typically diagnosed at later stages than in many other countries [28] and around 55 per cent do not receive any active anti-cancer therapy [29]. For modelling, I presume that 45 per cent of patients presenting symptomatically receive the same treatment as those whose cancers have been screen-detected, whilst the remainder receive only palliative care. Palliative care for lung cancer has been costed at £3,000 per patient [30]. Thus, the expected costs of treating cancers which present symptomatically are [(0.45)(£12,000) + (1-0.45)(£3,000)] = £7,050.

### Test parameters

The yield of a screening programme is influenced by the sensitivity and specificity of the CT screening test, and by the prevalence of cancer in the target population. The published estimates of the sensitivity of a single CT screen vary considerably, from around 55 per cent to over 90 per cent. The lower values tend to be reported by studies at their earliest phases and they therefore probably represent results taken from "high on the learning curve". The realistic minimum using experienced testers appears to be around 80 per cent [11]. Although meta-analyses [14,31] of independent studies have produced averages of estimated test sensitivity in excess of 90 per cent, I shall use a more conservative value of 85 per cent. The same meta-analyses produced average specificities of around 83 per cent, although studies reporting more recently have cited specificities of between 93 and 97 per cent [11]. The false-positive rate reported in a Mayo Clinic sample after five years [32] also implies a specificity within this range. It is probable that improving specificity over time can be explained by the accumulation of experience also. Again, I shall use a relatively conservative value, namely, a specificity of 85 per cent.

Lung cancer is a disease of the elderly. Only at the peak ages of presentation, from the mid-70 s onwards, does the incidence rate in England exceed 0.5 per cent. The screening debate, however, is rarely couched in terms of the general population. The subjects of all of the clinical studies of CT screening have being high-risk, selected using criteria such as age, occupation and history of cigarette smoking. The lung cancer prevalence rates reported in such studies range between 0.4 per cent and 13.6 per cent [33], with the USA studies typically reporting the higher prevalences [11]. As a basis for modelling a UK programme, I assume that the population targeted for screening will also be selected on the basis of risk, and will exhibit a prevalence of disease higher than that of the general population. I use a prevalence value of 1 per cent, the weighted average of the prevalences reported in three European CT screening studies, namely, those based in Germany [34], Ireland [35] and Italy [36].

### Health gains

**Figure 1:**
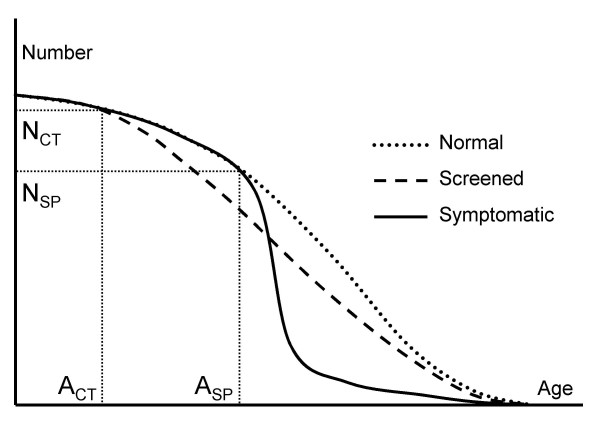
Cancer survivors over time.

I estimate LY gains from screening using a survival approach. Life Tables provide mortality rates and survival rates, by age and by sex. The mortality rate increases with age, and the Life Table data enable the plotting of a survival curve, which maps the number surviving from a cohort of individuals at any given age, *A*. In Figure 1, the curve labelled "Normal" pertains to a cohort whose members are subject to all of the normal causes of death; this is the curve produced using the Life Table data directly. To estimate an individual's life expectancy at any chosen age, *A*, we calculate the number of "years alive" in the cohort at each particular age. Summing the "years alive" from *A *to the oldest possible age in the Life Table gives the total number of years lived by cohort members from age *A*. In effect, this is the area under the survival curve. The expectation of life at age *A *is then obtained by dividing the total number of years lived by the number in the cohort alive at age *A *[37].

Now consider a cohort whose members are destined to contract lung cancer. Prior to presentation, the cohort will decline in numbers as for the "Normal" cohort. Following presentation, the current evidence from the UK suggests that most will die within the one or two years immediately following diagnosis and treatment. Mortality rates for those who do survive will eventually revert to those appropriate to a normal cohort. This cohort is modelled as "Symptomatic" in Figure 1, with presentation occurring at age A_SP_. Again, the area under the curve represents the total number of years lived by that cohort.

Finally, consider a cohort destined to contract cancer, but where the cancer will be detected by pre-emptive CT screening at age A_CT_. Any intervention entails an increased mortality risk with the result that, initially, the relative decline in numbers in this cohort will be greater than the relative decline of those in either a normal or a pre-symptomatic cancer cohort. However, presuming that earlier detection will indeed offer improved longer-term survival, the relative decline will be lower than for the symptomatic cohort subsequently (as represented by "Screened" in Figure 1). From the geometry of Figure 1, it is apparent that the LY gains from screening would fall were (i) survival rates following symptomatic presentation to improve, (ii) survival rates following screen-detection to decline, (iii) lead time, (A_SP_-A_CT_), to increase.

With respect to the calculations, the "Normal" curve uses UK Life Table data for males, estimated for the years 2003–5 [38]. I model survival in a male, as opposed to female, cohort, for two reasons. First, for any given age at intervention, males face a shorter life expectancy and, second, lung cancer is more prevalent amongst males. For a cancer cohort, mortality rates beyond A_SP _are modified using rates derived from Cancer Registry survival data. At present, 1-year survival following diagnosis and treatment in the UK is around 22 per cent, falling to around 6 per cent at 5 years [39]. For those presenting in their 60 s, the rates are more favourable, at around 27 and 8 per cent, respectively [40]. Data from the USA indicate that survival continues to fall beyond 5 years, although at an appreciably gentler rate [41]. This pattern of initially-high mortality, quickly tapering off, suggests a negative exponential formulation for mortality rate. For "Symptomatic", I define the mortality rate at ages *A *after presentation (*M*_*A*_) as:

*M*_*A *_= 0.7*(*A *- A_SP_)^-1.3 ^(*A *- A_SP_) = 1, 2, 3,....

The estimated rates are applied up to the age at which the normal mortality rate exceeds the estimated rate and, thereafter, the rate following symptomatic presentation defaults to the normal rate. This function actually over-states survival somewhat, as it implies 1-, 5-, and 10-year survival rates of 30, 14 and 11 per cent, respectively.

As regards long-term survival following CT screen detection within a clinical trial, the most authoritative data reported thus far are from the ELCAP investigators [42]. They report a 1-year survival rate of around 95 per cent, with a 10-year survival of 80 per cent. This result is corroborated by a reported 10-year survival of 83 per cent for a mobile screening programme in Japan [43]. These data form the basis of our "Screened" survival curve, and we model the mortality rate following screen-detection as:

*M*_*A *_= 0.1*(*A *- A_CT_)^-0.8 ^(*A *- A_CT_) = 1, 2, 3,....

As before, the estimated rates are applied up to the age at which the normal mortality rate exceeds the estimated rate and, thereafter, the rate following screen detection defaults to the normal rate. This function under-states post-screening survival as reported, because it implies 1-, 5- and 10-year survival rates of 90, 77 and 70 per cent, respectively.

The optimal age at which to screen remains to be established. As the survival model was informed principally by the ELCAP data, I set the age at screening in the model to the reported average age of those screened in that study, namely, 61 years. There exists no evidence as to when such cancers would have presented symptomatically although, given they are occurring in a high-risk population, presentation would presumably occur earlier than in the general population. Those involved in the European CT screening trial and in the ELCAP study [44,45] have conjectured that lead time could be 4–8 years. I used the value least favourable to the screening scenario, namely, 8 years.

NICE guidance [46] requires that health outcomes be discounted at the same interest rate as that used for discounting costs, namely, at 3 1/2 per cent per annum. In addition, NICE requires health gains to be expressed as quality-adjusted life years (QALYs), to facilitate comparisons with other health care interventions. Longitudinal research on quality of life following treatments for lung cancer is meagre. Quality of life seems to be poor in the immediate post-treatment phase but improves thereafter, especially amongst the longer-term survivors [47]. Evidence of health state utilities following treatment comes only from cases of symptomatic presentation although it might be expected that, were cancer to be detected and treated at the earliest stages, the health state utilities of patients would be noticeably higher. I used a rounded value for the quality adjustment coefficient identified for symptomatic presenters, namely, 0.6 [48-50], implying that 1 LY gained = 0.6 QALYs gained.

## Results

The survival model predicts that males in the general UK population can expect, when aged 61 years, to live a further 19.8 years, i.e. until 80–81 years of age. It predicts that those individuals destined to present with cancer symptomatically will live a further 10.9 years, i.e. until around the age of 71–72 years. With an assumed lead time of 8 years, this estimated age at death implies a mean survival after symptomatic presentation of nearly 3 years. Extrapolating the UK's 5-year UK survival data to 10 years, mean survival after presentation is actually 1.9 years. As intended, the model over-states post-presentation survival.

The model predicts that individuals whose cancers have been detected by screening at the age of 61 will survive a further 16.7 years, dying at the age of 77–78 years. Having a cancer which is detected and treated at the earliest stages, therefore, costs the individual 3.1 years of normal life. Compared with symptomatic presentation, however, the health gains per cancer detected at screening are 5.7 LYs (subtraction not exact owing to rounding). The absence of trial evidence makes independent validation of this result impossible. Perhaps the best that can be said is that, if we are willing to presume that screen-detected cancers will typically be at stage 1, whilst symptomatic ones will present at stage 3 or 4, then this gain is consistent with the difference in median survival times of 4–6 years reported in the Mayo Clinic series [51]. Given the assumed translation between life years and QALYs, the expected health gain per cancer screen-detected equals 3.4 QALYs. These QALYs are obtained over a period of up to forty years following screening, and the discounted gain is 1.7 QALYs.

The survival model generates two results necessary to complete the treatment cost calculation, equation (2). First, it specifies the lead time, E = 8. Second, between the ages of 61 and 69, the Life Table data predict a decrease in the number of survivors, (N_CT_-N_SP_), of 12 per cent, i.e. C = 0.88. Given that we have already specified R = 3 1/2 per cent, G_CT _= £12,000 and G_SP _= £7,050, it follows that T = £7,286.

Values for all the parameters in equation (5) have now been specified. Substitution into the equation produces an expected incremental cost per person screened of £201. This comprises the test cost of £60, £75 expended on investigating the false positives, and £66 expended on investigating and treating the true positives, net of expected treatment costs following symptomatic presentation. The expected incremental benefit per person screened equals 5.3 quality-adjusted life days, and the incremental cost effectiveness ratio amounts to £13,910.

### Sensitivity analysis

The consequences of changing values for the sensitivity and specificity parameters can be traced through equation (5). As noted earlier, some of the clinical studies have reported values in excess of 90 per cent for both. Allowing the sensitivity parameter to increase by 5 percentage points (from 85 to 90 per cent) increases the number of true positive test results. A higher yield from screening is accompanied by higher costs, although the net effect is to lower the ICER to £13,392. This represents a fall of around 4 per cent from the baseline estimate. An equivalent rise in specificity reduces the number of false positives being sent for unnecessary investigation. Expected cost is reduced, there are no consequences for expected health benefit, and the ICER falls by around 12 per cent, to £12,186. The two changes combined produce a 15 per cent fall in the baseline ICER, to £11,764.

Other things remaining equal, the cost effectiveness of screening increases as the prevalence of cancer in the target population increases. A screening programme for a target population with a prevalence of 1.5 per cent, rather than the assumed 1 per cent, would have an ICER of £10,784, around 22 per cent lower than the baseline estimate. A prevalence of 2 per cent would produce an ICER of £9,221, 34 per cent lower than baseline. Contrariwise, the prevalence of lung cancer in the UK's general male population aged 60–69 years is considerably lower, at around 0.2 per cent. Screening with this level of prevalence in the target population yields an ICER of £51,424, nearly four times the baseline estimate and well above the NICE threshold for cost effectiveness.

NICE is conscious that unit costs can vary locally and the guideline [23] presents the ranges of values collected across different providers. The range for the CT scan is £50–103, suggesting that the cost of CT scanning could, under some conditions, be almost double that of the baseline estimate. Were we to assume that unit CT costs were indeed to be twice as high as our baseline value, i.e. £120 as opposed to £60, the estimated ICER would rise from £13,910 to £18,065, an increase of approximately 30 per cent. The guideline's upper bound for the cost of the bronchoscopy investigation is £721. Using this value in place of £503 increases the ICER by 17 per cent above baseline, to £16,280. Making both cost adjustments simultaneously increases the ICER by 47 per cent, to £20,434.

This having been said, it is perhaps more reasonable to expect that the unit cost of procedures in a specific screening context will be lower than when measured in general usage, owing to economies of specialisation. For example, a detailed analysis of the costs of flexible sigmoidoscopy employed in colorectal cancer screening clinics [52] produced a unit cost of less than one-half of the contemporary tariff price for sigmoidoscopy estimated in general settings [53]. Were the costs of investigation in lung cancer screening to follow the same pattern, the ICER for screening would fall. Halving the cost of investigation, from £503 to £252, reduces the estimated ICER from £13,910 to £11,182, a fall of around 20 per cent from baseline.

It is known that survival rates following lung cancer treatment in the UK are low in comparison with those achieved elsewhere in Europe. The reasons for this remain uncertain, although diagnostic delay and (non-)use of novel therapies have been advanced as explanatory factors. Countries such as France and the Netherlands achieve 1-year survival rates of around 40 per cent and 5-year rates in excess of 10 per cent [54]. Were the survival rates of symptomatically-presenting cancers in the UK to move towards these levels, it follows that the life year gains realisable from CT screening would fall correspondingly. The "French approach" can be simulated by using a different mortality rate function in the model for those who present symptomatically, namely:

*M*_*A *_= 0.6*(*A *- A_SP_)^-1.0 ^(*A *- A_SP_) = 1, 2, 3,....

This function implies 40 per cent survival at one year, and 17 per cent at 5 years. The model now predicts a smaller health gain from screening, namely, 5.4 LYs rather than the baseline 5.7 LYs. Obtaining superior survival following symptomatic presentation, however, must have resource implications. In France, 75 per cent of patients are in receipt of active, as opposed to palliative, treatment [55], compared with less than 50 per cent in the UK. To accommodate a higher proportion of patients in active treatment in the model, *G*_*SP *_can be re-specified as [(0.75)(£12,000) + (1-0.75)(£3,000)] = £9,750, which is higher that the baseline value of £7,050. The net effect of more treatment and better survival is an ICER for screening of £13,786, around 1 per cent lower than the baseline estimate. From the point of view of the cost effectiveness, therefore, the impact of the improved survival of symptomatic presenters which results from more treatment is counter-balanced by the increased costs of that treatment.

The estimate of health gain has been based on data for males, although it is a simple matter to replicate the screening regimen for women. Using the Life Tables for females we find that, age-for-age, modelled life expectancies for women exceed those of men for each of the three survival curves. Normal life expectancy for UK women at 61 years of age is 22.8 years, compared with men's 19.8, for example, and the model estimates female life expectancy for screen-detected cancer at 18.5 years, compared with 16.7 for males. For a women with lung cancer, the expected gain from screening is 7.0 LYs, compared with a man's 5.7 LYs. The female health gain translates into 2.0 discounted QALYs, and there is no reason to suppose that the costs of screening would differ between men and women. Other things remaining equal, the baseline ICER for female CT screening is £11,710, 16 per cent lower than the equivalent ICER for males.

The modelled screening regimen entails a single, prevalence, screen for each subject. It is nevertheless evident from the literature that some screening enthusiasts have contemplated regimens involving further rounds of screening. Whilst not set up to consider such regimens specifically, some consequences can be inferred from the model. For example, two CT scans separated in time by less than 12 months would serve to increase the sensitivity of the screening regimen although, almost inevitably, with some loss of specificity. Such a regimen would entail test costs of £120, as opposed to £60. If sensitivity was thereby increased to 90 per cent, whilst specificity fell to 80 per cent, the ICER for such a regimen would, according to our model, be £18,944, a 36 per cent increase over baseline. Reverting to the original scenario of a single screen at age 61, suppose a second round of screening were to occur 3 years later, at age 64, now with a lead time of 5 years. The model suggests that the prevalence of cancers available for detection in the second screen would have to be around 6.5 per 1,000 for the second round to be as cost effective as the first. It is not evident whether the disease progresses sufficiently rapidly to produce this rate, although it is clear that the cost effectiveness of a second round of screening will vary directly with effectiveness of the first.

Finally, two of the survival model's assumptions merit consideration. First, the cancer-related survival curves are departures from the general population survival curve, derived from the Life Tables. As the individuals being modelled are destined to succumb to lung cancer, it would seem inevitable that they are less healthy than the general population and must experience significant co-morbidity. To reflect this, the "Normal" survival curve should have been based on higher age-specific mortality rates, although independent Life Tables for this morbid population do not, of course, exist. A simulation which increases all mortality rates by age by an arbitrary 10 per cent for the model's "Normal" curve suggests a fall in expected QALY gain from 1.7 to 1.6. The ICER rises by 6 per cent, to £14,727. The net effect is actually quite small, because health gain is determined by the difference between the "Symptomatic" and the "Screened" curve and each depends on the same "Normal" curve.

Second, the assumed lead time in the model was set to the maximum currently deemed possible, although evidence could, of course, eventually reveal it to be shorter. If the lead time is halved – four years rather than the assumed eight – expected costs fall by around 5 per cent, owing to the costs of managing symptomatic presenters moving forward in time. A shorter lead time implies earlier deaths for the symptomatic cases, and the expected QALY gains from screening rise from 1.7 to 3.1. The ICER becomes £7,226, 48 per cent lower than the baseline estimate.

## Discussion

It is evident from the model that the appropriate selection of screening targets is crucial for efficiency. Indeed, it is unlikely that un-targeted screening could ever be justifiable economically. Progressively restricting the programme to higher-risk individuals improves the economic case for screening in two ways. First, it reduces the numbers eligible for screening, and thereby lowers overall programme costs. Second, the selection of higher-risk individuals implies higher prevalence in the target population, and higher prevalence reduces the ICER, other things remaining equal. The economic viability of a UK screening programme is therefore predicated on the screeners' ability to establish appropriate criteria for identifying targets. Some success in this respect has already been reported [56,57]. It is probable that criteria will include not only disease-related risk factors such as cigarette smoking and occupational exposure to carcinogens but also factors related to capacity to benefit from treatment after detection [58].

Earlier it was noted that, were age at screening to be the only consideration, it would be more cost effective to screen women than to screen men, although this calculation failed to allow for the lower disease prevalence typical amongst females. These conclusions will require reconsideration, if an anticipated growth in prevalence amongst females relative to males eventually materialises [59]. At present, when both age and prevalence are taken into account, it is more cost effective to screen men than it is to screen women. Within the model, screening women is as least as cost effective as screening men when female prevalence is at or beyond around 80 per cent of male prevalence.

The calculations in the model conform to NICE's current accounting conventions, as would seem appropriate for the UK (or, more accurately, for England). However, parochial accounting conventions limit the capacity to generalise from the results [60], and NICE conventions do differ somewhat from those of most other countries. In particular, cost effectiveness evaluation requires a definition of perspective, i.e. identifying those to whom the costs and benefits accrue. NICE requires that the government or NHS perspective be used in its evaluations, as it sees itself as being concerned with the best uses of the public health care budget [61]. Most other countries using evaluation results to inform policy consider the societal perspective to be the more appropriate i.e. the relevant costs and benefits are those which accrue to any member of the entire population [62].

The use of different accounting conventions can yield different conclusions. For example, social costs are likely to be higher than NHS costs, for four reasons. First, social costs encompass all NHS costs, by definition. Second, attending for screening inevitably entails the incurring of time and travel costs on the part of those being invited. These costs can be sizeable: in a trial of clinic-based screening for colorectal cancer, time and travel costs added 26 per cent to gross NHS costs of all detection and treatment [63]. Third, screening according to our model requires people to undergo treatment during their early-60 s (when they might be employed), rather than in their late-60 s (when they might have retired). It is possible, therefore, that society will incur net production losses from earlier detection, as a result of workers undergoing treatment. Fourth, the informal sector (family or charity) provides a considerable input to terminal care, above and beyond that provided by the NHS. As the estimated benefits of screening are the same under either evaluation perspective, it follows that screening will be less cost effective when judged from a societal perspective than when judged from an NHS perspective. The magnitude of all these additional costs are, at present, unknown, so no serious assessment can be made of the likely difference between the NHS-perspective and the societal-perspective ICERs.

NICE's current position requires it to follow UK Treasury guidance in discounting costs and benefits at the same rate of 3 1/2 per cent. Academic debate over the discounting of benefits nevertheless persists, especially in circumstances of prevention where life years might accrue only in the distant future [64,65]. Some economists have argued that QALYs are logically un-discountable or, if they are discounted, the rate should be lower than that used for costs. Indeed, the latter was NICE's own position until 2004. The effect of discounting benefits at a lower rate in our model is considerable. At a zero discount rate, unit health gains change from to 1.7 QALYs to 3.4, and the ICER falls to £6,873. Discounting benefits at a zero rate would enable the original ICER to be maintained even if social costs were twice NHS costs. In fact, doubling all costs and failing to discount benefits in the model produces an ICER of £13,746, almost identical to the baseline estimate. It should be noted that NICE's current conventions have attracted scientific criticism [66] and it is by no means clear that these conventions are sustainable over the longer term.

Although models can provide guidance in the face of limited evidence, they cannot overcome the evidence vacuum which characterises several important real-world aspects of screening. First, practically nothing is known currently about how individuals would respond to an invitation to participate in mass lung cancer screening, beyond the likelihood of expressing an interest in principle [67]. Willingness to be screened for cancer is predictable from individuals' characteristics and, ironically, individuals who smoke tobacco appear to be amongst those least inclined to engage in health promotion activities. Smoking, for example, is associated with non-participation in US cervical [68] and colorectal [69] screening. A survey of potential users of CT screening in the USA revealed that, compared with non-smokers, a significantly lower proportion of smokers would consider being screened or would opt for treatment if a cancer were to be screen-detected [70].

With respect to the cost effectiveness model, a low compliance rate is of little consequence. With all the costs in the model being variable, reducing participation in screening by, say, one half, halves the benefits obtained but it also halves the costs, and the ICER as calculated remains unchanged. The cost effectiveness of a real-world programme would be affected significantly by a low participation rate only if fixed costs (e.g. those of administration and management) were sizeable. The refusal of treatment following detection is more damaging to the case for screening. If a screened subject refuses treatment, s/he incurs screening costs to no benefit, thereby effecting an increase in the programme's ICER. Were 1-in-5 screen-detected cancers in the model to refuse treatment, the ICER would rise by 25 per cent. In a Japanese study [43], however, only 5 per cent of patients with screen-detected cancers were reported as having refused treatment.

Second, the absence of experimental data means we have no clear understanding of the future impact of lung cancer screening on risk behaviour, specifically, cigarette smoking. On the one hand, it has been argued that those attending for screening are particularly susceptible to smoking cessation interventions [71]. On the other, smokers might feel that opportunities for early detection of cancer reduce the incentive to curtail their risky activities. Third, positive screening results are likely to create psychological morbidity, irrespective of any subsequent survival gains, as has been demonstrated for cervical screening [72]. These remain unaccounted for in our lung cancer model, simply because the effect is unknown. Finally, it is probable that a CT screening programme would generate quantities of "incidentalomas" [73], that is, asymptomatic abnormalities other than lung cancer detected by serendipity. Again, the consequences for cost effectiveness are unpredictable. Whilst the detection of incidentalomas is often viewed negatively, in view of the risks of over-treatment or of creating anxiety, a positive consequence is also possible. A screening programme which leads to the cost effective treatment of detected abnormalities in addition to lung cancer is might well be producing greater health gain for less-than-proportionate cost, thereby making the screening programme even more cost effective than it nominally appears.

## Conclusion

Using a model whose parameters were specified by recourse to the available evidence, I modelled a plausible screening scenario with an ICER below the NICE threshold. Therefore, the answer to the question – could CT screening for lung cancer ever be cost-effective in the UK? – must be squarely in the affirmative. I stress that this model is offered as a prelude to obtaining experimental evidence, and not as a substitute for such evidence. This having been said, it follows that, were a future clinical/economic trial to reproduce the parameter values employed in this model, then a screening programme consistent with both model and trial would itself be cost effective.
